# Correlation of bone density to screw loosening in dynamic stabilization: an analysis of 176 patients

**DOI:** 10.1038/s41598-021-95232-y

**Published:** 2021-09-01

**Authors:** Hsuan-Kan Chang, Jason Ku, Johnson Ku, Yi-Hsuan Kuo, Chih-Chang Chang, Ching-Lan Wu, Jiing-Feng Lirng, Jau-Ching Wu, Wen-Cheng Huang, Henrich Cheng, Shih-Ming Hsu

**Affiliations:** 1 Department of Neurosurgery, Neurological Institute, Taipei Veterans General Hospital, Taipei, Taiwan; 2 College of Medicine, National Yang Ming Chiao Tung University, Taipei, Taiwan; 3 Department of Biomedical Imaging and Radiological Sciences, National Yang Ming Chiao Tung University, No. 155, Sec. 2, Li-Nong St., Beitou District, Taipei, 112 Taiwan, ROC; 4 University of California, Los Angeles, USA; 5 Department of Radiology, Taipei Veterans General Hospital, Taipei, Taiwan; 6 Institute of Pharmacology, National Yang Ming Chiao Tung University, Taipei, Taiwan

**Keywords:** Neurology, Neurological disorders, Musculoskeletal system

## Abstract

Although osteoporosis has negative impacts on lumbar fusion, its effects on screw loosening in dynamic stabilization remain elusive. We aimed to correlate bone mineral density (BMD) with screw loosening in Dynesys dynamic stabilization (DDS). Consecutive patients who underwent 2- or 3-level DDS for spondylosis, recurrent disc herniations, or low-grade spondylolisthesis at L3-5 were retrospectively reviewed. BMD was assessed by the Hounsfield Unit (HU) in vertebral bodies (VB) and pedicles with and without cortical bone (CB) on pre-operative computed tomography (CT). Screw loosening was assessed by radiographs and confirmed by CT. HU values were compared between the loosened and intact screws. 176 patients and 918 screws were analyzed with 78 loosened screws found in 36 patients (mean follow-up: 43.4 months). The HU values of VB were similar in loosened and intact screws (*p* = 0.14). The HU values of pedicles were insignificantly less in loosened than intact screws (including CB: 286.70 ± 118.97 vs. 297.31 ± 110.99, *p* = 0.45; excluding CB: 238.48 ± 114.90 vs. 240.51 ± 108.91, *p* = 0.88). All patients had clinical improvements. In conclusion, the HU values, as a surrogate for BMD, were unrelated to screw loosening in DDS. Therefore, patients with compromised BMD might be potential candidates for dynamic stabilization rather than fusion.

## Introduction

During the past decade, bone mineral density (BMD) reportedly has been assessed by measurement of the Hounsfield Unit (HU) on computed tomography (CT) scans in spinal surgery^1^. There is an increasing popularity of such quantification since the correlations between HU and BMD have been established^2,3^. The adaptation of the HU on pre-operative CT scans allows a swift evaluation of patients’ BMD and can serve as an alternative to dual-energy X-ray absorptiometry (DEXA), which has been a standard assessment for BMD pre-operation. Recent reports have demonstrated the application of the HU values in the prediction for the risks of pseudoarthrosis, instability of implants, and bone density-related complications in spinal fusion surgery^4,5^.

Dynamic stabilization has also gradually gained acceptance in the past decade as an option of surgical management for lumbar spondylosis^6–9^. In reports of short to mid-term follow-up, pedicle-screw based dynamic stabilization systems, such as the Dynesys (Zimmer Biomet, Warsaw, Indiana) dynamic stabilization (DDS), has demonstrated similar clinical success rates to the standard lumbar fusion surgery^9–11^. However, durability remains the most frequent concern of these non-fusion constructs, especially in patients with inadequate BMD. The fatigue failure at the interface between the bone and screws, which frequently would cause screw loosening, remains the biggest challenge for DDS due to the need for continuous movement in such motion-preservation device^12^. The incidences of screw loosening in DDS were approximately 5% per screw and 20% per patient in 2–5 years post-operation^6–8,11,13–16^, although there were no associated adverse effects on the clinical outcomes. Since the demands of mechanical strength and durability for these dynamic screws are likely higher than the conventional screws designed for fusion^17,18^, it is reasonable to infer that the better the BMD, the lower rates of screw loosening and the higher chances of clinical success in the dynamic stabilization surgery of the lumbar spine. Nevertheless, there are scant reports on the correlation of BMD and success of DDS.

Since it remains elusive in the literature how BMD would affect the success of DDS in lumbar spine surgery, the current study aimed to evaluate BMD by HU values and correlate with the incidences of screw loosening in a series of patients who underwent surgery. To date, this is the first study to address the correlation between HU and screw loosening in DDS.

## Methods

### Patient enrollment

A consecutive series of patients who underwent surgical decompression and stabilization with 2- or 3-level DDS at L3 to L5 from 2007 to 2015 were retrospectively extracted from a prospectively collected database for analysis. The inclusion criteria were symptomatic spinal stenosis with hypertrophic ligamentum flavum, facet hypertrophy, minimal or less than grade I spondylolisthesis, degenerative disc disease, and recurrence of herniated intervertebral discs (HIVD) that were refractory to medical treatment. The clinical manifestations included neurogenic claudication, radiculopathy, or low back pain. The exclusion criteria were Meyerding grade I or II spondylolisthesis, coronal or sagittal plane deformity, and osteoporotic vertebral fractures. Patients who did not complete the follow-up protocols for more than 2 years were also excluded from the study. All patients had posterior midline incisions for laminectomy and pedicle-screw based DDS stabilization through a paramedian fascia incision for screw insertion via the Wiltse approach. The surgical technique was identical to that detailed in our previous publications^6–8,16^.

The present study received institutional review board (IRB)/ethics approval in our institute (Taipei Veterans General hospital). All study procedures were performed in accordance with relevant guidelines/regulations. A waiver of informed consent was approved by the IRB.

### Clinical and radiological evaluations

The clinical outcome parameters were prospectively collected and reviewed retrospectively. The parameters included visual analog scales (VAS) for back and leg pain, Oswestry Disability Index (ODI) scores, and modified Japanese Orthopaedic Association (JOA) scores. Clinical data were collected by two special nursing assistants during admission at pre-operation, and post-operative 1.5, 3, 6, 12 and 24 months at outpatient clinics.

**Figure 1:**
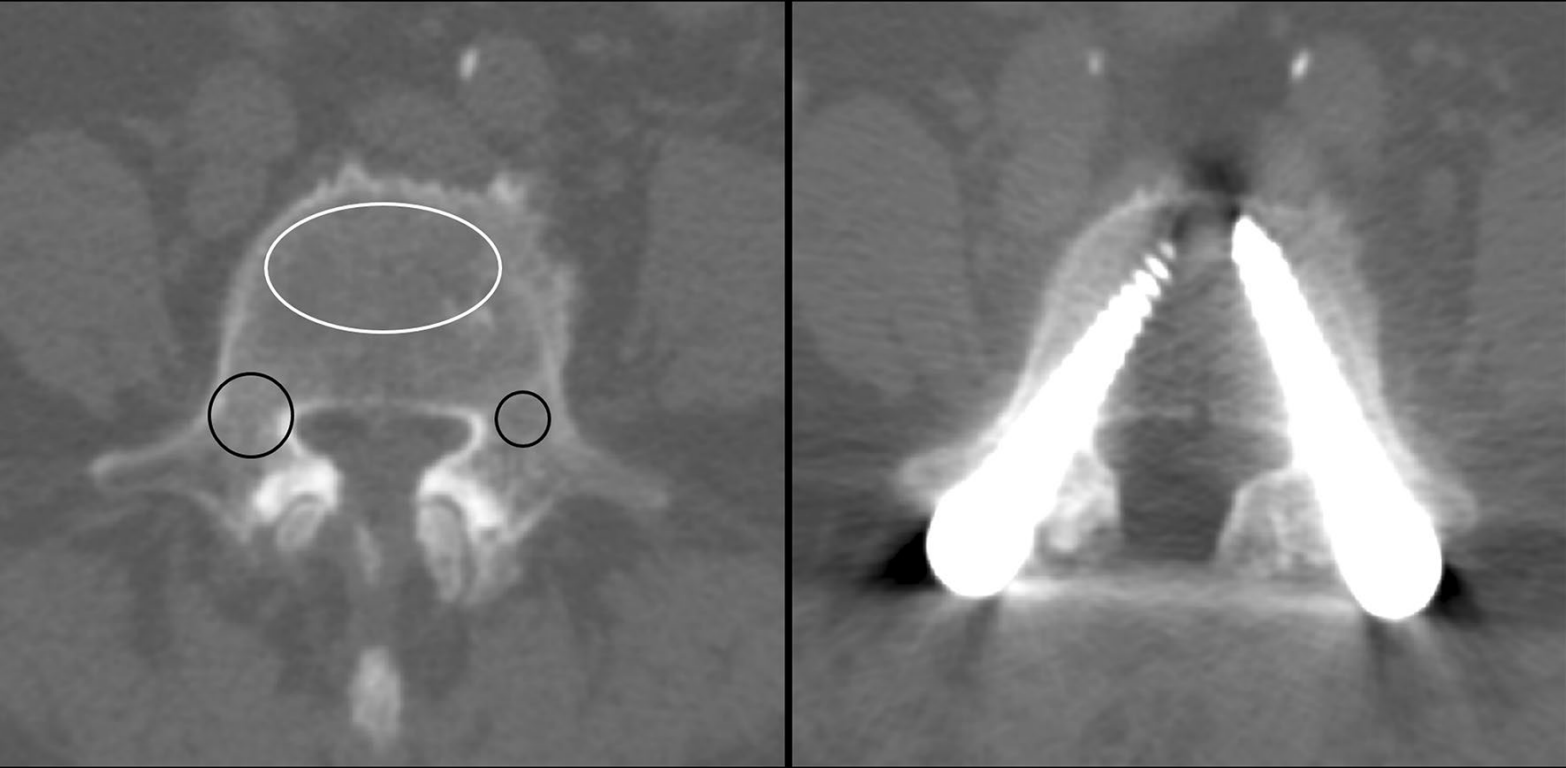
A 69 year-old female underwent Dynesys dynamic stabilization (DDS) system at L3–4–5. Left*:* the measurement of the CT Hounsfield Unit (HU) values of the L5 vertebral body (VB, white circle) and pedicles, including (large black circle at the right pedicle) and excluding the cortical bone (small black circle at the left pedicle) in this patient. The HU value of the VB was 124. The HU values of the right pedicle including and excluding the cortical bone were 206 and 156, respectively. The left HU values of the left pedicle including and excluding the cortical bone were 250 and 180, respectively. Right: although all HU values measured on pre-operative CT in this patient were lower than the mean HU values in our cohort, post-operative 24 months CT demonstrated no screw loosening at L5.

The Hounsfield Unit value was determined on pre-operative CT scans within 6 months prior to surgery in all patients. The region of interest (ROI) was calculated on PACS systems (SmartIris, Taiwan Electronic Data Processing Co.) with a similar area size. The HU value of the vertebral body was measured on an axial plane at the pedicle level where the screws’ tips were usually set (Fig. 1). The ROI of the VB HU value included only the trabecular bone and avoided the cortical bone (CB). The measurement of the HU values of the pedicle, including or excluding the CB, are also demonstrated on Fig. 1. The pedicle ROI was placed at the widest part of the pedicle on an axial plane where the screw trajectory usually goes through. We measured the HU at every VB and pedicle (both right and left pedicles, including and excluding CB) in every segment whenever there was a screw being placed. Therefore, each HU value, as a surrogate for BMD, corresponded to a certain screw and analyzed independently for its relationship to screw loosening.

**Figure 2:**
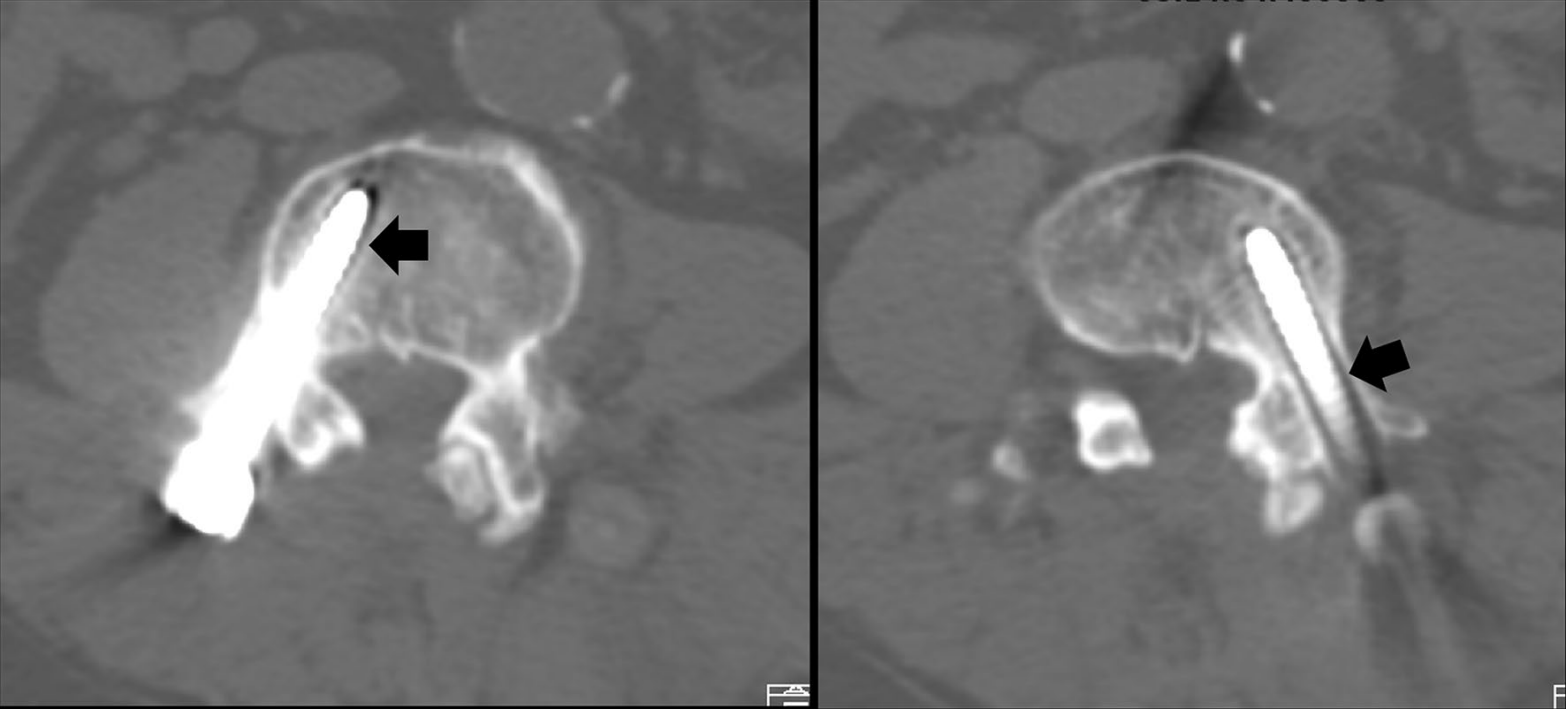
Post-operative 24 months CT demonstrated bilateral screw loosening at L4 in a 64 year-old male who underwent dynamic stabilization at L3–4 (black arrow: double halo sign), although all HU values of L4 pedicles were much higher than the mean HU values in our cohort. (L4 right pedicle HU including/excluding cortex bone: 350/276; left pedicle HU including/excluding cortex bone: 355/272; VB HU: 117).

Post-operative follow-up of radiographic films was undertaken at each time point in the outpatient clinic. Post-operative MRI and CT scans were arranged at approximately 18 to 24 months after surgery. Screw loosening was defined as a double-halo sign (radiolucency around the screw for more than 1 mm wide) on radiographic films or CT scans (Fig. 2). The radiological evaluation for screw loosening was performed by 2 independent radiologists on the PACS system.

### Statistical analysis

SPSS software (SPSS, Inc.) was used for all statistical analysis. Paired t-tests and independent t-tests were used for continuous variables. Chi-squared tests were done for categorical variables. Statistical significance was determined by a *p* value < 0.05.

### Ethics approval

IRB approved (Taipei Veterans General hospital IRB #2020-04-006AC).

### Patient consent

Waived by IRB.

## Results

### Demographic characteristics and clinical outcomes

Demographic data are shown in Table 1. A total of 176 patients undergoing 2- or 3- level pedicle screw-based DDS from L3 to L5, and a total of 918 screws were assessed in our study. There were a total of 78 loosened screws found in 36 patients; 840 screws were intact. The loosening rate was 8.4% (78 out of 918 screws) in all screws, and 20.5% (36 out of 176 patients) in all patients. The age at operation (61.80 ± 11.59 vs. 62.57 ± 11.79 years, *p* = 0.72), sex distribution (female 41.6% vs. male 50.7%, *p* = 0.33), and follow-up length (45.58 ± 19.32 vs. 42.83 ± 17.25 months, *p* = 0.22) were all similar between the loosening and non-loosening groups of patients. In regard to comorbidities, the number of smoking patients, or with hypertension or diabetes were not different between the two groups (*p* = 0.86, 0.93, 0.19 for smoking, hypertension, and diabetes, respectively).

**Table 1:** Patients’ demographic data.

	Loosening group	Non-loosening group	*p* value
Patient number	36	140	
Age (years)	61.80 ± 11.59	62.57 ± 11.79	0.72
Gender (M/F)	21/15	69/71	0.33
Follow-up (months)	45.58 ± 19.32	42.83 ± 17.25	0.22
Smoking	4 (11.1%)	17 (12.1%)	0.86
Hypertension	17 (47.2%)	65 (46.4%)	0.93
Diabetes	13 (36.1%)	34 (24.3%)	0.19

Values are presented as mean ± SD or the number of patients (%).

Collected clinical outcome parameters included VAS for back and leg pain, ODI scores, and modified JOA scores. The final follow-up data demonstrated significant improvement compared to pre-operative data in the entire cohort (*p* < 0.001 in all clinical parameters, Supplementary Table S1).

### CT Hounsfield Unit (HU) value in loosened and intact screws

**Figure 3:**
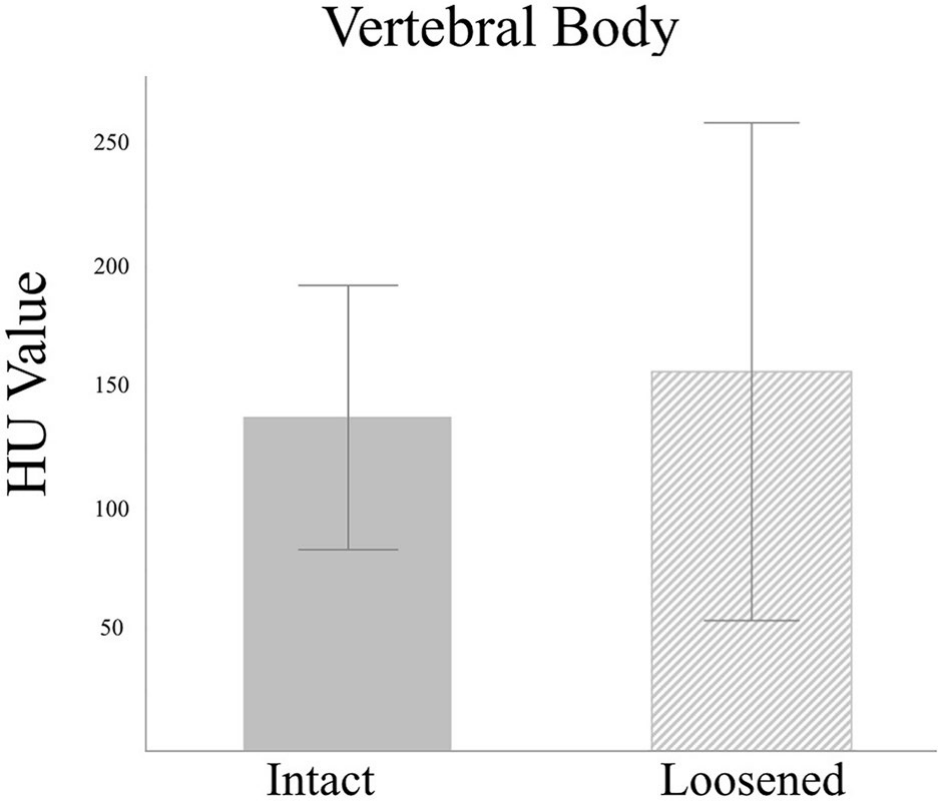
Mean HU values for the L3–5 vertebral bodies showed no difference between the loosened and intact screws. (154.83 ± 101.70 vs. 136.65 ± 54.53, *p* = 0.14).

**Figure 4:**
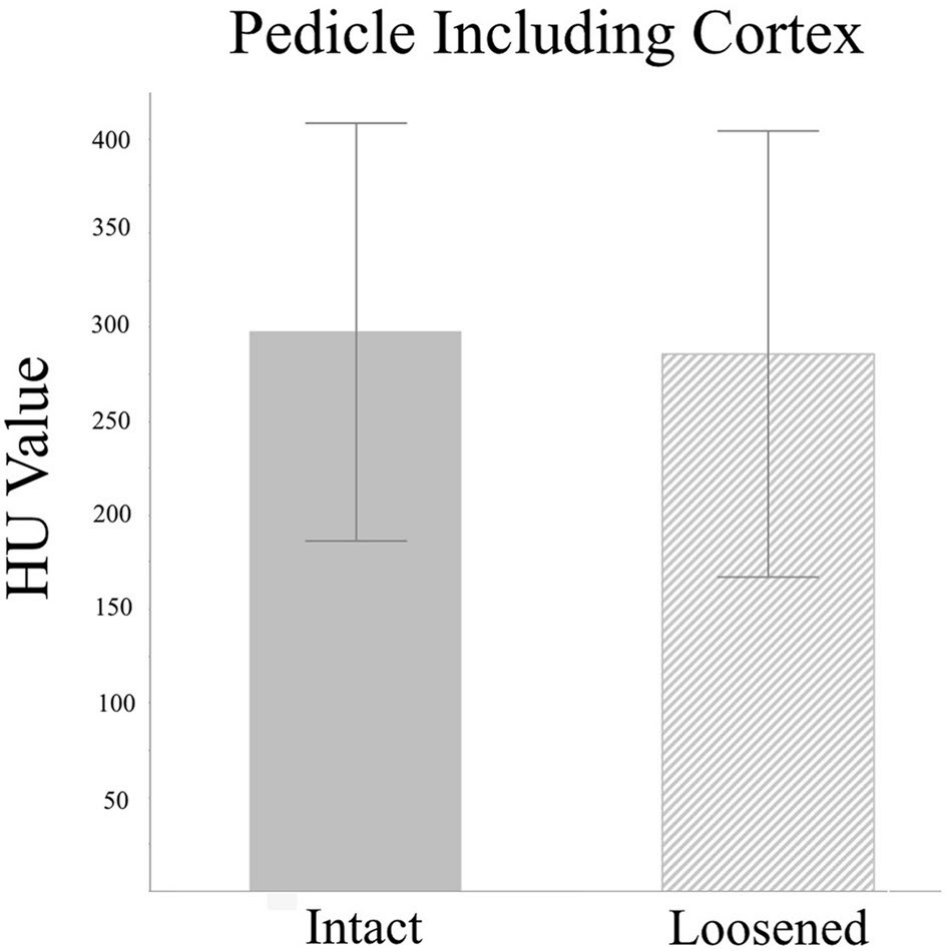
Mean HU values for the L3–5 pedicles including the cortical bone was insignificantly lower in the loosened screws compared to the intact screws. (286.70 ± 118.97 vs. 297.31 ± 110.99, *p* = 0.45).

**Figure 5:**
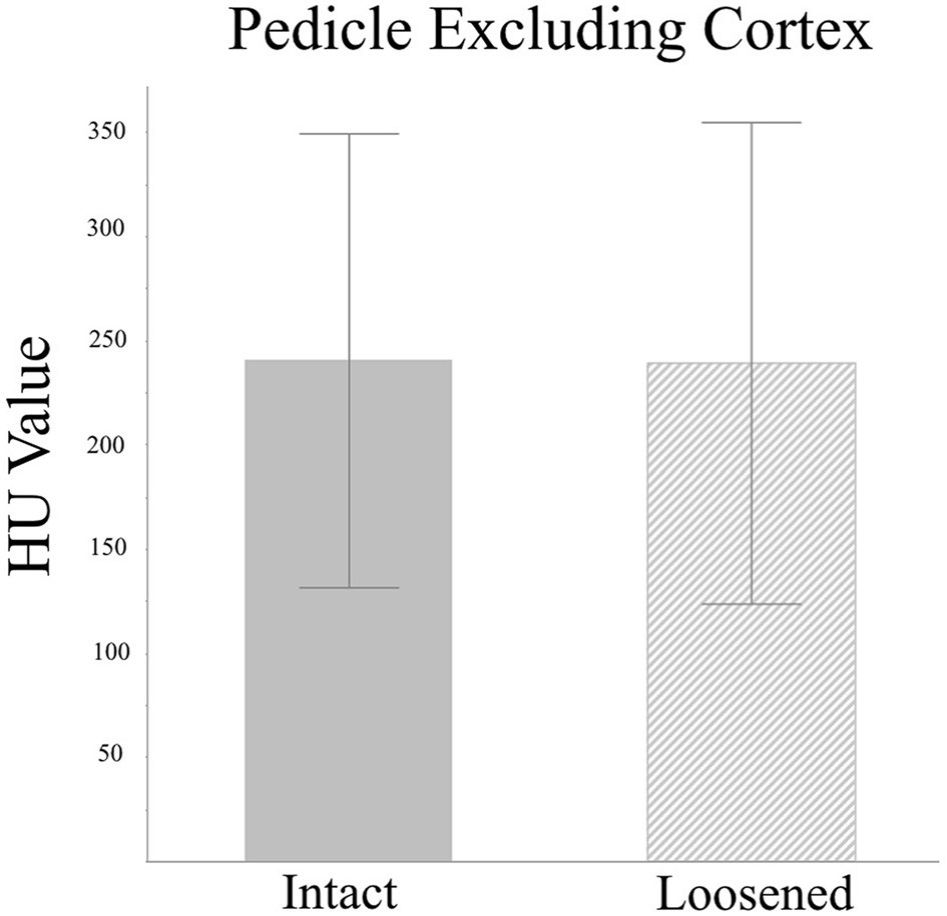
Mean HU values for the L3–5 pedicles excluding the cortical bone were insignificantly lower in the loosened screws compared to the intact screws. (238.48 ± 114.90 vs. 240.51 ± 108.91, *p* = 0.88).

The HU value was measured on every vertebral body and pedicle on pre-operative CT scans. The HU value of pedicles, including and excluding the CB, were also measured, respectively. The HU values of the VB demonstrated no significant difference between the loosened and intact screws (154.83 ± 101.70 vs. 136.65 ± 54.53, *p* = 0.14, Fig. 3). The HU values of pedicles, including CB and excluding CB, were lower in the loosened screws than the intact ones, but without statistical significance (including CB: 286.70 ± 118.97 vs. 297.31 ± 110.99, *p* = 0.45, Fig. 4; excluding CB: 238.48 ± 114.90 vs. 240.51 ± 108.91, *p* = 0.88, Fig. 5).

## Discussion

Our study examined 918 screws in a series of 176 patients who underwent pedicle-screw based DDS. The loosening rate was 8.4% (78 out of 918 screws) by screw, and 20.5% (36 out of 176 patients) by patient. We found that the VB HU was not significantly different between the loosened screws and the intact screws. The HU of pedicles, whether including or excluding CB, was also similar between the loosened screws and the intact screws. Our data suggested that BMD may not play an important role in screw loosening for dynamic stabilization.

Pedicle screw loosening is a common complication after lumbar fusion surgery, especially in osteoporotic patients. The incidence of screw loosening can reach 60% among osteoporotic patients in long-term follow-ups after pedicle screw placement^19,20^. It is widely accepted that osteoporosis has a huge impact on pedicle screw loosening, and screw loosening is associated with a high risk of revision in lumbar fusion surgery^21,22^. Screw loosening is also a well-known complication in pedicle screw-based dynamic stabilization. There were nearly 5% of screws found with loosening in about 20% of patients undergoing pedicle screw-based DDS in the existing literature. The risk factors included diabetes and old-age^7^. However, it has never been investigated if osteoporosis is a risk factor for screw loosening in pedicle screw-based DDS.

Dual energy X-ray absorptiometry (DEXA) is considered the standard method in assessing bone mineral density (BMD) to evaluate the severity of osteoporosis based on World Health Organization (WHO) criteria^23^. Although most academic spine surgeons have recommended the evaluation of BMD before spine surgery, less than 50% of them routinely arranged DEXA for their patients pre-operatively^24^. Alternative methods to evaluate BMD have been advocated in addition to DEXA. The Hounsfield Unit value, measured on computed tomography, is another common tool which can help in detecting osteoporosis in lumbar spine instrumentation^25^. Studies have shown that the CT HU value is closely related to BMD^26,27^. Several studies also demonstrated that the CT HU value is able to predict pedicle screw loosening after lumbar screw fixation^28,29^.

Our study is the first to adopted the CT HU value as a surrogate for BMD, and investigate the relationship between the CT HU value and screw loosening in pedicle screw-based DDS. We found that the CT HU value of VB is similar between loosened and intact screws (VB HU: 154.83 ± 101.70 vs. 136.65 ± 54.53, *p* = 0.14) in pedicle screw-based DDS. Some authors have advocated that there may be a stronger association between HU values of pedicle and screw loosening than HU values of VB in lumbar fusion surgery^30,31^. Due to a more significant relevance of biomechanical stability and a stronger trabecular architecture, there are reports suggesting that screw stability depends more on the pedicular part than the VB^32,33^. Our data showed different findings: that neither the VB CT HU nor the pedicle CT HU value was related to screw loosening in pedicle screw-based DDS (pedicle HU including CB: 286.70 ± 118.97 vs. 297.31 ± 110.99, *p* = 0.45; excluding CB: 238.48 ± 114.90 vs. 240.51 ± 108.91, *p* = 0.88), although the loosened screws had insignificantly lower HU. However, screw loosening did not lead to worse clinical outcomes in our patients undergoing DDS, which is compatible to our previous research^7^.

There were 78 loosened screws found in 36 patients in our study. The loosening rate was 8.4% (78 out of 918 screws) in total screws, and 20.5% (36 out of 176 patients) in total patients. The loosening rate was slightly different from our published data (around 5% loosening rate in total screws, and 20% in total patients)^6–8,16^, probably because the S1 screws were not included in the present study. The measurement of the CT HU value at the sacral bone has rarely been reported in the existing literature. There is no widely acceptable method of measuring VB and pedicle HU at the sacral bone so far. Therefore, the patients with S1 screws were not enrolled in our cohort. Risk factors for screw loosening in pedicle screw-based DDS were old-age and diabetes^6,8^. The mean age was similar and the number of diabetic cases was not different in our two groups. Although smoking has a negative effect on lumbar fusion surgery, Kuo et al. reported that smoking led to a trend of more screw loosening in pedicle screw-based dynamic stabilization, but with no statistical significance^16^. However, in the present study the number of smoking cases was larger in the non-loosening group, without statistical difference between the two comparative groups. In summary, the real cause of screw loosening in dynamic stabilization remains unknown. Large prospective studies and multivariate analysis may be able to answer the question.

The primary strength of our study is that we have measured the CT HU value at every VB and pedicle occupied by screws. Approximately 170 patients, 900 screws and all relevant CT HU values were measured and analyzed. A comprehensive data set of CT HU values was acquired in our cases, compared to previous studies that only measured VB or pedicle CT HU values at a certain segment as a representative for all segments of HU values when both BMD and HU values can be very different at each segment among one patient^25,28,29^. Therefore, it is more precise to say that the CT HU value, as a surrogate for BMD, is not associated with screw loosening in our patients undergoing pedicle screw-based DDS.

There were also limitations to our study. First, this is a retrospective study from a cohort of patients who underwent dynamic stabilization. Further prospective studies are warranted to validate our findings. Second, although the CT HU value and DEXA T-scores are common tools for detecting osteoporosis or osteopenia in the lumbar spine, the ideal definition and quantification method for the bone quality of the lumbar spine remain undetermined. The measurement of the CT HU value may not be a perfect reflection of bone quality, but is still currently an acceptable proxy for assessing osteoporosis or osteopenia.

## Conclusion

In this series of DDS, the clinical outcomes were not affected by screw loosening. The pre-operative CT HU values of the VB and pedicles were not different between the loosened and intact screws. Also, the HU values, as a surrogate for BMD, were not correlated with screw loosening in DDS. Therefore, patients with compromised BMD might be potential candidates for dynamic stabilization.

## Supplementary Information

Supplementary Information.
