# Supplementary material for: Correlation of bone density to screw loosening in dynamic stabilization: an analysis of 176 patients
Source: Sci Rep. 2021 Sep 1;11:17519. doi: 10.1038/s41598-021-95232-y (PMC8410763; doi:10.1038/s41598-021-95232-y)
Supplement: Supplementary file 1 — Supplementary Information. [file 41598_2021_95232_MOESM1_ESM.docx]

Table S1. Clinical outcomes

|  | Pre-operation | Final Follow-up | p-value |
| --- | --- | --- | --- |
| VAS of back pain | 5.42 + 3.10 | 3.26 + 2.96 | **p<0.001** |
| VAS of leg pain | 6.4 + 2.87 | 2.37 + 2.77 | **p<0.001** |
| ODI | 24.54 + 8.80 | 10.71 + 8.84 | **p<0.001** |
| Modified JOA scores | 5.91 + 8.22 | 9.96 + 3.78 | **p<0.001** |
| Values are presented as mean ± SD. Boldface type indicates statistical significance. VAS: visual analog scale. ODI: Oswestry disability index. JOA: Japanese Orthopaedic Association. | | | |
